# How Can We Prevent a Resurgence of Vaccine Nationalism?

**DOI:** 10.34172/ijhpm.9038

**Published:** 2025-05-18

**Authors:** Qi Shao

**Affiliations:** Department of Situation and Policy, Huaibei Normal University, Huaibei, China.

## Introduction

**Figure 1:**
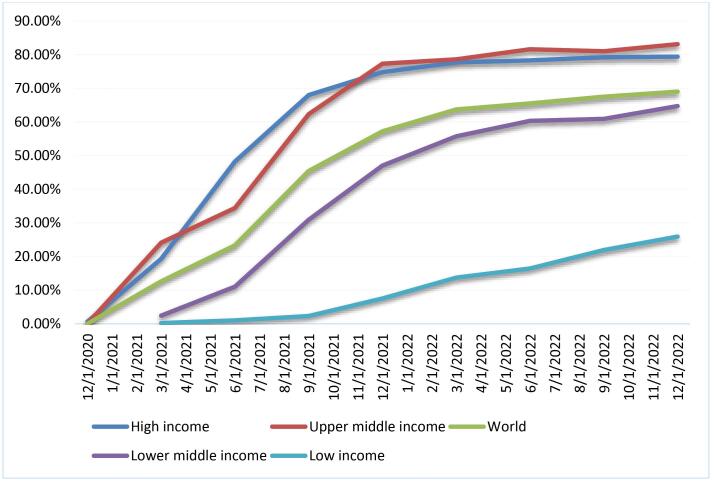
Share of People Who Received at least One Dose of COVID-19 Vaccine. (Data = Total number of people who received at least one vaccine dose/the country’s total population). **Source:** Our World in Data. Share of People Who Received at least One Dose of COVID-19 Vaccine. https://ourworldindata.org/grapher/share-people-vaccinated-covid?facet=none&country=High+income~Low+income~OWID_WRL~Lower+middle+income~Upper+middle+income. Data as of December 30, 2022.

 In the early days of the COVID-19 pandemic, the competition among vaccine-producing countries (as well as rich countries that do not produce vaccines) to secure early access to vaccines and to prioritise the vaccination of their populations, which made it more difficult for low- and middle-income countries to access vaccines and created an “immunisation gap” between countries of different income (Figure 1). This “my country first” ideology of global vaccine distribution has been termed “vaccine nationalism” by Bollyky and Bown.^1^ The international community has been strongly criticising vaccine nationalism. The World Health Organization (WHO) Director-General Tedros Adhanom Ghebreyesus even described it as a catastrophic moral failure.^2^ However, no country has yet apologised for vaccine nationalism, let alone promised to correct it. This means that if we do not find effective ways to eliminate vaccine nationalism, it may resurface in the next pandemic.

## Ethical Evaluation of Vaccine Nationalism

 Before discussing how to prevent a resurgence of vaccine nationalism, we must first ask a key question: is vaccine nationalism immoral? If the answer is yes, then all we have to do is continue to strengthen our critique of vaccine nationalism and try to persuade all countries to abandon the formulation and implementation of vaccine nationalism policies. If the answer is no, we must find another way to eliminate vaccine nationalism. But the answer may not be a clear yes or no.

 From a medical ethics perspective, the vaccine distribution should aim to minimise severe illness rates and mortality and protect the medical system. Therefore, those at highest risk from COVID-19, such as healthcare workers, the elderly and those with co-morbidities, should be prioritised for vaccination, regardless of their nationality or where they live. Vaccine nationalism has undermined this medical ethical principle and exacerbated inequities in global vaccine distribution, and is therefore immoral.^3^

 However, the impact of the pandemic goes far beyond health. In response to the COVID-19 pandemic, countries have taken varying degrees of measures to prevent and control the pandemic, including but not limited to the closure of factories and schools, traffic blockades, large-scale nucleic acid testing, and social distancing restrictions, which have led to serious social problems such as unemployment, school dropouts, hunger, violent conflicts, and economic downturns, putting enormous pressure on countries. The sooner a country establishes herd immunity, the sooner it can restore social order and economic vitality and reduce the loss of life. Therefore, the global distribution of vaccines is not just a medical issue but a political issue of vital interest to all countries.

 From a political ethics perspective, vaccine nationalism does not violate moral principles. The international community is an anarchic “self-help system,” “those who do not help themselves, or who do so less effectively than others, will fail to prosper, will lay themselves open to dangers, will suffer.”^4^ As the main actors in the international community, countries must actively fight for their interests to survive and develop. Moreover, the legitimacy of representative governments and leaders also depends in part on their ability to protect citizens’ interests, including their health interests. Therefore, without exception, all countries will actively seek to vaccinate their populations as soon as possible. In other words, vaccine nationalism is an ideology shared by countries rather than an ideology of individual countries.

 Some may disagree with this view, as some countries have explicitly condemned vaccine nationalism. For example, on May 14, 2020, leaders of some low- and middle-income countries, along with heads of international organisations, former heads of government and experts, signed an open letter stating: “Access to vaccines and treatments as global public goods are in the interests of all humanity. We cannot afford for monopolies, crude competition and near-sighted nationalism to stand in the way,” and “guarantees COVID-19 vaccines, diagnostics, tests, and treatments are provided free of charge to everyone, everywhere. Access needs to be prioritised first for front-line workers, the most vulnerable people, and for poor countries with the least capacity to save lives.”^5^ However, as Hans Morgenthau put it, “the actor on the political scene cannot help ‘playing an act’ by concealing the true nature of his political actions behind the mask of a political ideology.”^6^ The real motivation for these countries to jointly sign the open letter may not be to oppose vaccine nationalism, but precisely to implement it, because if vaccines are distributed as they advocate, they may access vaccines earlier than other countries, including vaccine-producing and wealthy countries.

## Conditions for the Rise of Vaccine Nationalism

 Although vaccine nationalism is an ideology shared by all countries, it can prolong the pandemic, increase the global death toll and threaten the global health system and economy by prioritising the vaccination of the entire population at home, including low-risk groups, over high-risk groups abroad.^1^ Hence, we still need to eliminate it. To do so, we must first understand how vaccine nationalism arises, and then we can apply the right medicine. Based on previous experience, we believe that the rise of vaccine nationalism requires the simultaneous fulfilment of three conditions.

 Condition a: Vaccines are scarce. In a pandemic, vaccine shortages force countries into a zero-sum game, triggering fierce competition for limited vaccines. Once supplies are adequate, vaccines become ordinary commodities, and vaccine nationalism naturally subsides. During the COVID-19 pandemic, vaccine nationalism peaked in 2021, the first year after the vaccine became available. After that, as production of the COVID-19 vaccine soared and the natural infection rate of the world’s population increased, the supply and demand for vaccines gradually balanced out, and some high-income countries even had a surplus of vaccines.^7,8^ Vaccine nationalism thus lost its foothold.

 Condition b: Health issues escalate into national security issues. The prevention and treatment of most infectious diseases is simply a health issue. Only a pandemic with high infectivity and mortality can cause severe social panic, economic decline, and political crisis and become a national security issue that the health sector cannot handle. Once the government, rather than the health sector, takes the lead in responding to pandemics, they may prioritise vaccination of their populations on the grounds of maintaining national security, and various vaccine nationalism policies will inevitably be introduced.

 Condition c: An endemic disease escalates into a pandemic. Not all infectious diseases are global. If an infectious disease, such as diphtheria, Ebola hemorrhagic fever, or yellow fever, spreads mainly in certain parts of the world,^9^ the demand for vaccines against it is also regional. There is no need for countries outside the epidemic area to vaccinate their populations. On the contrary, when an infectious disease goes from endemic to pandemic, the demand for vaccines skyrockets, and vaccine nationalism is likely to emerge.

**Figure 2:**
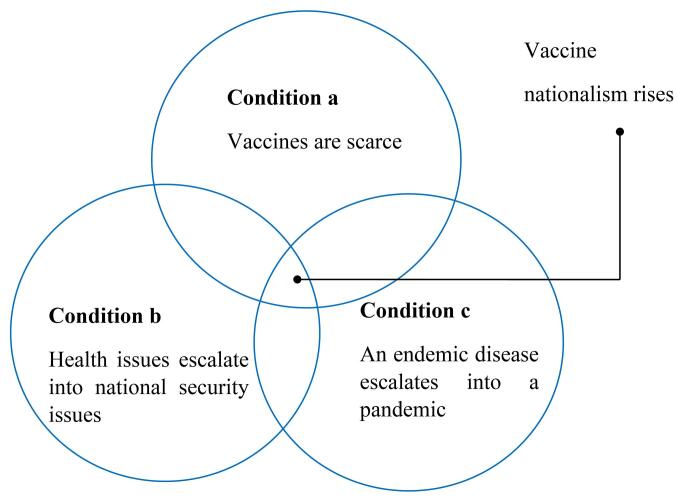
Model of the Rise of Vaccine Nationalism.

 The three conditions for the rise of vaccine nationalism are indispensable. In the absence of condition a, there is no need for countries to compete for vaccines; in the absence of condition b, governments will not interfere in the global distribution of vaccines, and medical professionals will use their expertise and medical ethics to decide who should be vaccinated first; in the absence of condition c, vaccines will be a necessity only for certain countries or regions, and will not trigger global competition for vaccines. That is, vaccine nationalism can only be triggered by a lack of vaccines in a pandemic that is highly infectious, has a high mortality rate, and has a global impact (Figure 2).

## Recommendations for Preventing a Resurgence of Vaccine Nationalism

 Once we understand the conditions for the rise of vaccine nationalism, we only need to remove one of these conditions to prevent a resurgence of vaccine nationalism.

 1. Accelerating the development of advanced vaccine technologies to improve vaccine production efficiency. Thanks to collaboration between scientists and huge government investment, the development of the COVID-19 vaccine took less than a year, a miracle in the history of vaccines.^10^ Cutting-edge mRNA technology has made outstanding achievements in this process, creating a new era of vaccinology with powerful platform technologies and a new model for vaccine development andplaying an important role in accelerating the production of COVID-19 vaccines.^11,12^ Further research into mRNA technology to improve vaccine production efficiency is key to preventing the resurgence of vaccine nationalism in the next pandemic. But even then, the period of vaccine shortage in a pandemic crisis can only be shortened, not eliminated. Because we cannot maintain crisis levels of vaccine production in non-crisis times – it is not politically or economically sustainable.

 2. Supporting the development of the vaccine industry in low- and middle-income countries and promoting vaccine research and development and production localisation. As Adar Poonawalla, chief executive officer of the Serum Institute of India (the world’s largest vaccine manufacturer), said: “Almost every country now wants to set up local manufacturing so that it never has to scramble for vaccines again.”^13^ However, vaccine research and production are challenges for most low- and middle-income countries. They need strong support from the international community. Some progress has been made in this area in recent years. In June 2021, South Africa, with support from the WHO, the Medicines Patent Pool, and the Act-Accelerator/COVAX, began establishing the mRNA Vaccine Technology Transfer Hub, which will share technology and technical expertise with local producers.^14^ In the future, at least 15 mRNA (vaccine) production sites in low- and middle-income countries will be established around this hub.^15^ However, the initiative also faces several risks, including uncertain funding, the threat of patent litigation by established mRNA vaccine manufacturers, and a range of governance issues.^16^ In addition, as the COVID-19 vaccine market shrinks dramatically and health development assistance continues to decline, low- and middle-income countries will need to find new business models to maintain mRNA vaccine production capacity during non-crisis periods. There may be value in using mRNA technology to develop and produce vaccines against other diseases.

 3. Strengthening drug development, production and promotion to reduce pandemic mortality. During the COVID-19 pandemic, the use of the commonly used drug dexamethasone and the new drug remdesivir reduced the risk of patient death, which played an important role in reducing the panic caused by COVID-19 in the international and national communities.^17-19^ Nevertheless, drugs for a pandemic may be hoarded by some countries, as happened in the COVID-19 pandemic,^20^ and intellectual property rights and know-how may be monopolised by a small number of pharmaceutical companies, preventing rapid global availability. However, we do not support governments forcing pharmaceutical companies to disclose patents or implementing “compulsory patent licensing” policies, as this would seriously undermine their incentive to innovate. If pharmaceutical companies are unwilling to donate their intellectual property rights and know-how, high-income countries or charities should purchase them from pharmaceutical companies and then make them available free of charge to low- and middle-income countries, which may be an effective way to alleviate the tension between incentives for innovation and access.

 4. Optimising the global epidemic prevention and control system to prevent or delay the spread of the epidemic. In the daily work of epidemic prevention and control, countries should establish and improve the sentinel surveillance system for infectious diseases, use big data, artificial intelligence, and other technologies to track the development of the epidemic in real time, and identify potential risks in a timely manner. After an outbreak, countries should promptly issue travel warnings and strengthen customs inspections and quarantines. Countries with an epidemic should actively work on traceability and maintain transparency of epidemic information. Only through sincere cooperation between countries can we prevent or slow the spread of the epidemic and buy time for vaccine research and development and mass production.

## Conclusion

 In an international society characterised by “anarchy” and “self-help,” all countries would compete to vaccinate their populations against a pandemic. Therefore, vaccine nationalism is an ideology shared by all countries, and we cannot eliminate vaccine nationalism by condemnation and persuasion but only by removing the conditions that it rises. As long as we remove any of these, we can prevent vaccine nationalism from resurfacing.

## Ethical issues

 Not applicable.

## Conflicts of interest

 Author declares that he has no conflicts of interest.

## Disclaimer

 I solemnly promise that I submit the paper completed independently by me and never plagiarize. I ensure that the results of other people’s data are properly cited with indicated sources. If this paper involves intellectual property disputes, I am fully responsible.
